# ASPDH inhibits the proliferation, migration, and invasion of liver cancer cells by regulating lactate metabolism and the NF-κB/PD-L1 pathway

**DOI:** 10.1016/j.clinsp.2026.100946

**Published:** 2026-05-13

**Authors:** Ding Li, Muzi Li, Mengfan Yan, Yuanyuan Xiong

**Affiliations:** a Department of Interventional Radiology and Vascular Surgery, Hunan Provincial People's Hospital (the First Affiliated Hospital of Hunan Normal University), Changsha, Hunan, China; b Department of Neurosurgery, The Second Affiliated Hospital of Nanchang University, Nanchang, Jiangxi, China

**Keywords:** Liver cancer, Lactate metabolism, ASPDH, PD-L1, NF-κB

## Abstract

**Background:**

Lactate has been proven to be an important metabolite involved in tumor initiation, progression, and tumor microenvironment, affecting patient prognosis. The role of lactate metabolism-related genes in liver cancer is unclear.

**Methods:**

The authors identified lactate metabolism-related genes from five key pathways and scored their relevance. Genetic and clinical data from TCGA-LIHC and validation cohorts (GSE54236, ICGC, and GSE116174) were analyzed to confirm survival associations. Sodium lactate rescue experiments were performed on ASPDH-overexpressing cells to investigate the role of lactate. *In vivo* xenograft models verified ASPDH’s effects on tumor growth and NF-κB/PD-L1 signaling.

**Results:**

From 320 lactate metabolism-related genes, four core genes were identified: LPCAT1, TMEM220-AS1, ASPDH, and LECT2. Low ASPDH expression correlated with poorer survival across multiple datasets. PD-L1 ROC analysis showed high diagnostic efficacy (AUC = 0.8). ASPDH knockdown enhanced proliferation, colony formation, migration, and invasion, while overexpression had opposite effects. Sodium lactate rescue experiments further demonstrated that exogenous lactate partially restored cell proliferation, migration, and invasion abilities suppressed by ASPDH overexpression, along with increased nuclear p65 and PD-L1 expression, confirming lactate's role in mediating ASPDH-regulated NF-κB pathway activity and PD-L1 expression. GSEA linked ASPDH to PD-L1 expression and checkpoint pathways. *In vivo*, ASPDH overexpression suppressed tumor growth, decreased nuclear p65 and PD-L1, and increased cytoplasmic p65, alongside reduced lactate secretion and NF-κB/PD-L1 modulation.

**Conclusion:**

ASPDH inhibits liver cancer cell proliferation, migration, and invasion by downregulating lactate secretion and suppressing the NF-κB/PD-L1 signaling pathway.

## Highlights

• Lactate metabolism genes are closely linked to hepatocellular carcinoma prognosis.
• Bioinformatics identifies ASPDH as a key lactate metabolism gene.
• ASPDH effectively inhibits the proliferation, invasion, and migration of liver cancer cells.
• ASPDH suppresses liver cancer progression via lactate metabolism and NF-κB/PD-L1 signaling.

## Introduction

Liver cancer is one of the most common malignant tumors in the world. It was the third leading cause of cancer-related deaths globally.^1^ Liver cancer patients are not sensitive to conventional radiation and chemotherapy.^2^ While lots of patients also do not benefit from sorafenib treatment.^3^ So, find the new treatment strategies to prolong the survival time for patients. However, given the significant heterogeneity of liver cancer patients, new prognostic markers are needed.

It was first described in the 1920s that even under aerobic conditions, tumor cells consume large amounts of glucose and secrete lactate, known as the Warburg effect, a hallmark of cancer.^4^ There is a close relationship between lactate and immunity. Lactate in the high glycolytic tumor microenvironment promotes the expression of Programmed Cell Death protein-1 (PD-1) in regulatory T-cells, leading to partial resistance to PD-1 blockade therapy.^5^ Bioinformatics analysis has made significant progress in many fields.^6^ The role of lactate-related genes in bioinformatics analysis of liver cancer remains to be further studied.

Aspartate Dehydrogenase (ASPDH) is a key enzyme that catalyzes the oxidative dehydrogenation of L-aspartate to produce oxaloacetate and NAD(P)H.^7^ Studies have shown that ASPDH relies on NAD/NADP to catalyze the oxidation of L-Asp and participates in the biosynthesis of Poly-3-Hydroxybutyrate (PHB) precursors by regulating gene clusters related to PHA synthesis.^8^ The ASPDH from Achromobacter denitrificans has been utilized in a biocatalytic system coupled with Glucose Dehydrogenase (GDH) for the efficient synthesis of L-2-aminobutyric acid (achieving a conversion rate of 97.2%).^9^ Additionally, aspartate β-semialdehyde dehydrogenase (ASADH), an analogous enzyme to ASPDH, has been identified as a potential target for antibacterial and herbicide drug design due to its crucial role in the synthesis of essential amino acids in microorganisms.^10^ Currently, there is still a lack of systematic research on the regulatory mechanisms, structure-function relationships, and liver cancer-related roles of ASPDH across different species. As a pivotal metabolic enzyme, the multifunctional biological characteristics of ASPDH warrant further in-depth investigation to uncover its potential in both fundamental metabolisms.

In the present study, genes related to lactate metabolism were obtained through 5 lactate pathways. Based on TCGA LIHC data, ssGSEA was executed to obtain LM. Enrichment score. WGCNA analysis, survival random forest, and LASSO analysis were used to screen for core genes. The authors aimed to explore the role of lactate metabolism-related genes in liver cancer.

## Material and methods

### Data collection

The Fragments Per Kilobase Million (FPKM) data for Liver Hepatocellular Carcinoma (LIHC), including 365 tumor samples, were downloaded from The Cancer Genome Atlas (TCGA) data portal (https://portal.gdc.cancer.gov/). The validation datasets GSE54236 and GSE116174 were obtained from the GEO database (https://www.ncbi.nlm.nih.gov/geo/), and the ICGC data were acquired from the ICGC Data Portal (https://dcc.icgc.org/). Additionally, the somatic mutation spectrum and relevant clinical information for LIHC were also obtained from the TCGA data portal.

### Screening of lactate-related genes

A search for the keyword 'lactate' in the Molecular Signatures Database v7.4 (MSigDB; https://www.gsea-msigdb.org/gsea/msigdb) identified five lactate-associated pathways: GOBP_LACTATE_METABOLIC_PROCESS, HP_INCREASED_SERUM_LACTATE, HP_LACTIC_ACIDOSIS, HP_LACTICACIDURIA, and HP_SEVERE_LACTIC_ACIDOSIS. From these pathways, the authors extracted 320 lactate metabolism-related genes and analyzed their expression profiles using the limma package in R.

### Screening of core genes and analysis of prognostic characteristics

From the initial 320 lactate phenotype-related genes, prognostic markers were further screened. Univariate Cox regression analysis identified 105 lactate-associated genes significantly linked to prognosis (p < 0.05). These genes were then subjected to single-sample Gene Set Enrichment Analysis (ssGSEA) to derive the Lactate Metabolism Enrichment (LM.Enrichment) score. Next, weighted gene co-expression network analysis (WGCNA) was performed on the TCGA dataset to identify gene modules correlated with the LM Enrichment score. The black module, exhibiting the strongest positive correlation, contained 148 genes. Subsequent univariate Cox regression analysis (p < 0.05) further refined this set to 77 prognostic genes. WGCNA Soft Threshold = 5. For patient stratification in Kaplan-Meier analysis, the authors adopted the “optimal cutoff” method implemented via the R package survminer. Moreover, the authors have included survival analyses of ASPDH across multiple validation sets (GSE54236, ICGC, and GSE116174).

### Random survival forest (RSF)

To further refine the prognostic markers among the 77 lactate-related genes, RSF analysis was performed. RSF extends random forests to censored survival data, handling high-dimensional genomic datasets while accounting for nonlinear relationships and interactions between genes. Genes were then ranked based on their Variable Importance (VIMP) in predicting patient survival outcomes. Genes with lower predictive importance were iteratively eliminated, reducing the candidate gene pool from 77 to 27. This step retained only the most biologically and prognostically relevant genes, minimizing interference.

### Least absolute shrinkage and selection operator (LASSO)

After obtaining the 27 candidate genes, LASSO regression was applied for final marker selection. As a penalized regression method, LASSO performs variable selection and model regularization simultaneously by imposing an L1-penalty constraint. A 10-fold cross-validation was used to determine the optimal penalty parameter (λ), balancing prediction accuracy and model complexity. Through rigorous screening, a core prognostic signature consisting of four genes: LPCAT1, TMEM220-AS1, ASPDH, and LECT2. Correlation analysis was performed between the immune checkpoint, clinical staging, and the lactate-related gene ASPDH. Hub genes within co-expression modules were identified using these stringent criteria: Module Membership (MM) > 0.8 (absolute value). Gene Significance (GS) > 0.6 for the primary clinical trait. Top 10% connectivity (kWithin) within the module. Additional filtering by differential expression (adj.p < 0.05) where applicable.

### ROC analysis

To evaluate the predictive performance of ASPDH expression in distinguishing PD-L1 immunotherapy responders from non-responders, the authors performed Receiver Operating Characteristic (ROC) curve analysis using the following approach: Gene expression data (ASPDH levels) and corresponding clinical response labels were extracted from the immunotherapy cohort. The ROC curve was constructed by plotting sensitivity against 1-specificity across a series of ASPDH expression cutoffs, with the Area Under the ROC Curve (AUC) calculated to quantify diagnostic accuracy. Statistical analysis included estimation of 95% Confidence Intervals for the AUC using DeLong's method, with all computations performed in R (v4.3.0) using the pROC package.^11^

### Drug sensitivity calculation

The drug sensitivity was analyzed by oncoPredict. The drug sensitivity prediction was performed using the GDSC2 database (Genomics of Drug Sensitivity in Cancer, v2022) as the reference pharmacology database. The ASPDH expression data related to drug sensitivity and drug sensitivity data were prepared. The ASPDH expression data were preprocessed and standardized, and the drug sensitivity data were integrated and cleaned. Gene features related to drug sensitivity were selected, and feature selection algorithms were used for screening. A machine learning model or other predictive model was established to predict drug sensitivity using the selected gene features as inputs. The model was evaluated and optimized through cross-validation or other validation methods to ensure its robustness and reliability. The trained model was used to predict drug sensitivity for new samples, yielding drug sensitivity prediction results.

### Analysis of mutation and functional enrichment associated with ASPDH

Somatic mutation profiles were compared between high and low ASPDH expression groups using the maftools package. The genes associated with ASPDH were analyzed by GSEA and KEGG pathway enrichment, and the results were obtained using the clusterProfiler tool.

### Cell culture

Hep3B (AW-CCH035), MHCC-97H (AW-CCH088), HepG2 (AW-CCH604), and HCC-LM3 (AW-CCH210) were cultured in MEM medium (iCell-0012, Icell, China) containing 10% FBS and 1% double antibodies. LX2 cells (AW-CCH088) were cultured in DMEM medium (iCell-0001, Icell, China) containing 10% FBS and 1% double antibodies. HepG2 was a low-metastatic liver cancer cell line, representing an early-stage/low-aggressiveness liver cancer phenotype, and was used as a control to assess the role of genes in tumor malignant transformation. Hep3B was a moderately to highly metastatic liver cancer cell line with intermediate metastatic potential, exhibiting greater invasiveness than HepG2. HCC-LM3 and MHCC-97H were highly metastatic liver cancer cell lines with strong invasive and metastatic potential. All cells were purchased from Abiowell Biology LTD.

### Cell grouping

Logarithmically growing cells were divided into six groups: Control, oe-NC, oe-ASPDH, si-NC, si-ASPDH#1, and si-ASPDH#2. Control group: Hep3B and MHCC-97H cells without any transfection treatment. oe-NC group: Transfected with the pcDNA3.1 empty vector. oe-ASPDH group: Transfected with an ASPDH-overexpressing plasmid to induce ASPDH upregulation. si-NC group: Transfected with a scrambled siRNA (with no homology to human genes) as a negative control for siRNA interference. si-ASPDH#1 and si-ASPDH#2 groups: Transfected with two different ASPDH-specific siRNAs targeting distinct regions of ASPDH mRNA. All plasmids were purchased from HonorGene. In brief, the cell transfection procedure was as follows: Two of the four sterile centrifuge tubes were taken, and each tube was filled with 95 µL serum-free MEM/DMEM medium. Then, 3 µg of oe-ASPDH, si-ASPDH#1, si-ASPDH#2 and 5 µL of Lip2000 (11,668,019, invitrogen, UK) were added separately to each tube. Following ASPDH overexpression, sodium Lactate (Lac) intervention (10 mM, 24 h)^12^ was performed on the specified groups to investigate the functional interaction between ASPDH and lactate. oe-ASPDH + Lac Group: Following transfection with the ASPDH-overexpressing plasmid, cells were treated with 10 mM sodium lactate for 24 h. oe-NC and si-NC were also added to the respective centrifuge tubes using the same method. Gently mix, let stand at room temperature for 5-min, then mix the two tubes gently. Finally, evenly add the mixed solution to the transduction wells and mix well. After culturing at 37 °C for 6 h in the incubator, fresh complete growth medium was replaced.

### qRT-PCR

Total RNA from cells was extracted using TRIzol (15,596,026, Thermo Fisher Scientific, Waltham, MA, USA). The reaction conditions were denaturation at 95 °C for 10-min, denaturation at 94 °C for 15 s, annealing at 60 °C for 30 s, for 40 cycles. The internal reference primer was β-actin. The primer sequences are shown in Table 1. With 2 μg cDNA as template, the relative quantitative method (2^-△△Ct^ method) was used to calculate the relative transcription level of the target gene: △△Ct = △ experimental group -△ Control group, △Ct = Ct (target gene)-Ct (β-actin).

**Table 1:** Primer sequences.

Gene	Sequences (5′−3′)
ASPDH	Forward primer: GAAAGGTAGCAAGTCAAGGCG
	Reverse primer: GACACGAAGGCTCAGGAGAT
LPCAT1	Forward primer: CATGAGGCTGCGGGGATG
	Reverse primer: GGGAAGAGCGTCAGTGTCAT
TMEM220-AS1	Forward primer: CCTTGCTCCAAGTCCCCTTC
	Reverse primer: GGGACTCCCGTGTTCATGTT
LECT2	Forward primer: CCAATGAGATCCGGACGTGT
	Reverse primer: TCCTGGCCCACAATCATTCC
β-actin	Forward primer: ACCCTGAAGTACCCCATCGAG
	Reverse primer: AGCACAGCCTGGATAGCAAC

### Western blot

Following cell collection and pre-processing, nuclear and cytoplasmic proteins were fractionated using Nuclear and Cytoplasmic Protein Extraction Kit (P1201, Applygen, China), while total protein was extracted from cells using the RIPA Kit (r0010, Solarbio, China). Protein samples were then subjected to immunoblotting with the following primary antibodies: rabbit anti-p65 (1:1000, 66,535-1-Ig, Proteintech), rabbit anti-PD-L1 (1:1000, AWA58022, Abiowell), rabbit anti-PCNA (1:5000, 10,205-2-AP, Proteintech), and mouse anti-β-actin (1:5000, ab8226, Abcam) as a loading control. Membranes were washed three times (10-min each) with PBS-Tween (0.1%) between incubations.

### Cell counting kit-8 (CCK-8)

Cells from each group were digested and counted, then seeded at a density of 1 × 10^4^ cells/well in a 48-well plate, 300 μL per well. After adhering to the wall, the cells were treated according to the above method for the corresponding time. Then, 30 μL of CCK-8 (NU679, Tongren, Japan) was added to each well, prepared by diluting the CCK-8 solution in complete growth medium, and the drug-containing medium was removed before adding 300 μL of medium containing CCK-8 to each well. After continuing to culture at 37 °C with 5% CO_2_ for 4 h, the absorbance (OD) at 450 nm was analyzed using a microplate reader, and the average value was plotted in a bar graph.

### 5-Ethynyl-2′-deoxyuridine (EdU)

EdU solution (C10310, Ruibio, China) was diluted in cell culture medium at a ratio of 1000:1 to prepare 50 μM EdU medium. Subsequently, each well was treated with 50 μL of 4% paraformaldehyde (N1012, NCM, China). Next, 50 μL of 2 mg/mL glycine was added to each well, and the glycine solution was discarded. After removing the glycine solution, cells were immediately processed for fluorescent staining and microscopic observation.

### Transwell

All sterile consumables (pipette tips, EP tubes) and extracellular matrix components (Matrigel, Corning) were equilibrated at 4 °C overnight prior to experimentation. 500 μL of 10% fetal bovine serum complete medium was added to the lower chamber. The membrane was then removed. Staining with 0.1% crystal violet (G1062, Solarbio, China) was performed for 5-min, followed by 5 washes with water. The membrane was placed on a glass slide for imaging.

### Cloning experiment

Single-cell suspensions were plated in 6-well culture plates at a density of 200 viable cells per well in 2 mL of complete medium. Plates were gently agitated to ensure uniform cell distribution before incubation at 37 °C in a humidified 5% CO_2_ atmosphere for 14‒21 days, with medium replenishment every 3-days. Cells were stained with 1 mL of staining working solution, followed by slow washing with running water and air-drying. Clones were counted.

### Lactate measurement

Lactate was measured according to the steps of the lactate assay kit (A019–2, Nanjing Jiancheng Bioengineering Institute, China). After mixing the sample with the reagents, it was allowed to react at 37 °C for 10 min. 2 mL of stop solution was then added and mixed, and the absorbance of each tube was measured at a wavelength of 530 nm with a 1 cm light path. Finally, the concentration of lactate was calculated using a standard curve and the calculation formula.

### Xenograft tumor model in nude mice

The subcutaneous xenotransplantation model of BALB/c nude mice was adopted to evaluate the inhibitory effect of ASPDH overexpression on the growth of liver cancer. Stably transfected Hep3B cells (1 × 10^6^ cells/mouse) carrying either oe-NC or oe-ASPDH were suspended in Matrigel and subcutaneously injected into the right flank of nude mice. Tumor growth was monitored by measuring the Length (L) and width (W) every 3-days, with tumor volume calculated using the formula V = W^2^ × L/2. After 35-days of observation, mice were euthanized, and tumors were harvested for further analysis. This study was approved by the Medical Ethics Committee of Hunan Provincial People's Hospital (No. [2024]−69).

### Statistical analyses

Graphpad Prism 8.0 software was used for statistical analysis. One-way ANOVA with Tukey's post hoc test was used for comparison among multiple groups. Statistical comparisons between groups across multiple time points were performed using two-way analysis of variance (ANOVA), followed by Bonferroni post hoc tests for multiple comparisons. A significance level of p < 0.05 indicated statistical significance.

## Results

### Lactate metabolism and screening of prognosis-related genes in liver cancer

**Fig. 1:**
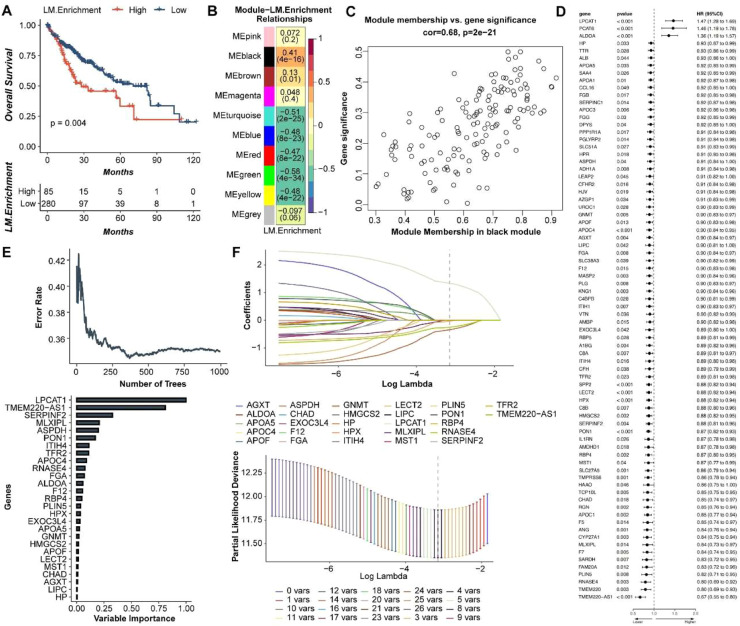
Screening of lactate metabolism-related genes associated with the prognosis of liver cancer. (A) Survival analysis based on LM.Enrichment score. (B) WGCNA analysis: correlations between modules and LM.Enrichment score. (C) Correlation between members of the Black module and gene significance with LM.Enrichment score. (D) Univariate Cox analysis. (E) RSF plot. (F) LASSO analysis.

A total of 320 lactate-related genes have been collected from 5 lactate-related pathways, and Cox analysis identified 105 lactate-related genes associated with the prognosis of liver cancer patients. LM.enrichment score was generated using ssGSEA with these genes. Survival analysis showed a statistically significant difference between the survival times of liver cancer patients with High and Low LM Enrichment score (p = 0.004) (Fig. 1A). WGCNA analysis revealed correlations between modules such as Module eigengene (Me)pink, Meblack, Mebrown, and LM Enrichment score. In the Meblack module, there is a high correlation between gene significance and module membership (Fig. 1B and 1C). Further screening of 178 lactate-related genes in the Meblack module was conducted by univariate Cox analysis, which identified 77 statistically significant lactate metabolism-related genes associated with the prognosis of liver cancer patients (p < 0.05) (Fig. 1D). RSF analysis reduced the genes to 27 (Fig. 1E). LASSO analysis further narrowed down to 4 core genes: LPCAT1, TMEM220-AS1, ASPDH, and LECT2 (Fig. 1F). In conclusion, lactate metabolism-related genes were closely linked to the prognosis of liver cancer patients.

### Survival analysis of lactate metabolism-related core genes

**Fig. 2:**
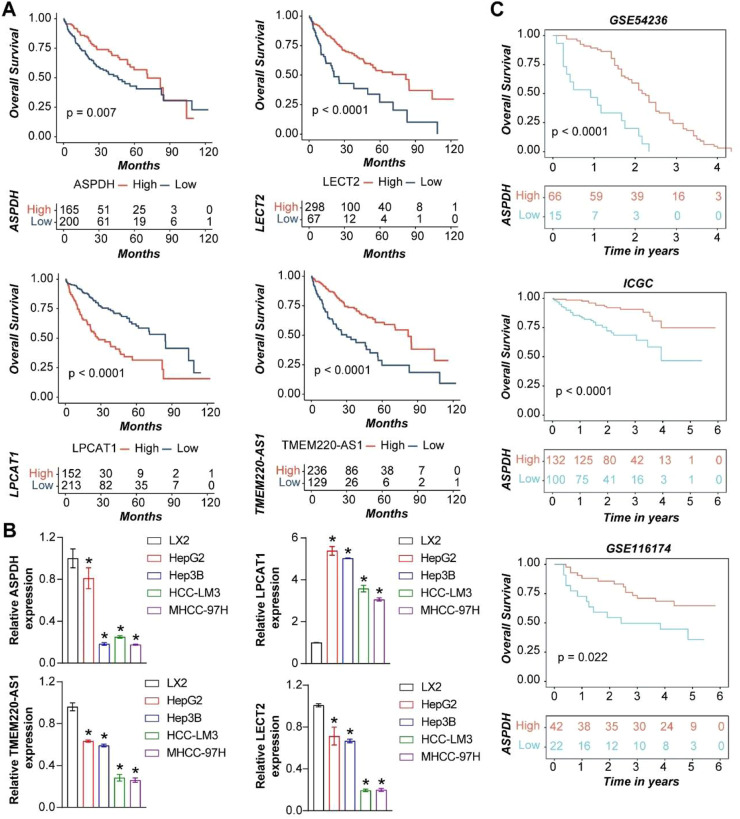
Prognostic analysis of lactate metabolism-related core genes. (A) Survival analysis of 4 lactate metabolism core genes (LPCAT1, TMEM220-AS1, ASPDH, and LECT2) in liver cancer. (B) The expression of LPCAT1, TMEM220-AS1, ASPDH, and LECT2 in five cell lines. (C) Survival analysis of ASPDH. * Indicates p < 0.05 compared to the LX2 group.

The above results identified 4 lactate metabolism-related genes (LPCAT1, TMEM220-AS1, ASPDH, and LECT2). Survival analysis showed a statistically significant association between the expression levels of LPCAT1, TMEM220-AS1, ASPDH, LECT2, and the survival time of liver cancer patients. Lower expression levels of TMEM220-AS1, ASPDH, and LECT2 were associated with lower 10-year survival rates in liver cancer patients, while lower expression of LPCAT1 was associated with higher 10-year survival rates (Fig. 2A). To investigate the relationship between liver cancer and the expression of these genes *in vitro*, the authors examined the expression levels of LPCAT1, TMEM220-AS1, ASPDH, and LECT2 in LX-2, HepG2, Hep3B, HCC-LM3, and MHCC-97H cell lines. Compared to LX-2, the expression of TMEM220-AS1, ASPDH, LECT2 significantly decreased in other cells, while LPCAT1 expression increased (Fig. 2B). The survival analysis of ASPDH in the validation sets (GSE54236, ICGC, and GSE116174) showed that lower ASPDH expression was associated with poorer survival rates (Fig. 2C). This indicated that the expression of these 4 genes is indeed abnormal in liver cancer cells.

### ASPDH inhibited the proliferation, invasion, and migration of liver cancer cells

**Fig. 3:**
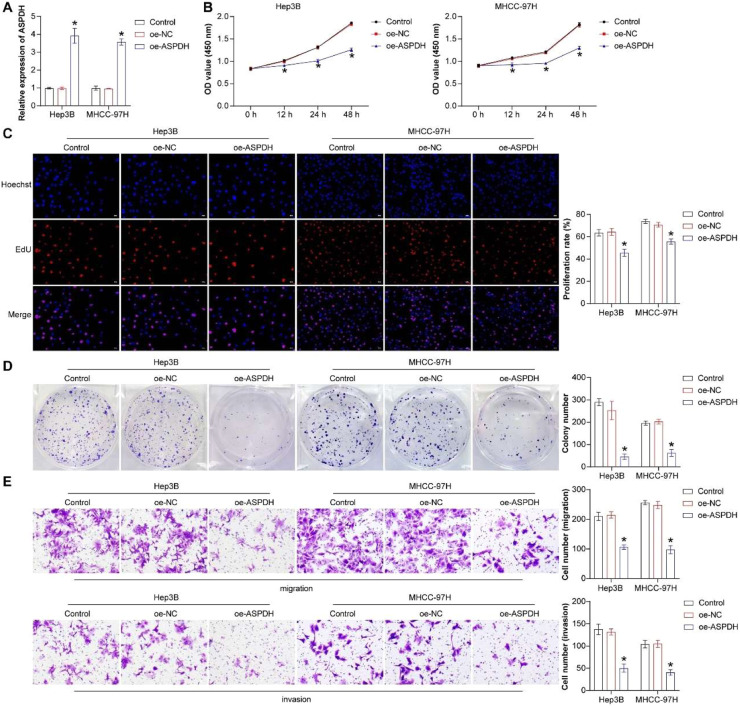
Overexpression of ASPDH could inhibit the proliferation, invasion, and migration of liver cancer cells. (A) The expression of ASPDH. (B) Cell proliferation. (C) EdU was used to analyze cell proliferation. (D) Clone assay was used to analyze cell proliferation. (E) Transwell analysis was used to measure cell migration and invasion. * Indicates a significance level of p < 0.05 compared to the oe-NC group.

LPCAT1, TMEM220-AS1, and LECT2 have been studied in liver cancer,^13^, ^14^, ^15^ so ASPDH was selected for further analysis. Based on the expression of ASPDH in 5 cell lines, the low-expressing Hep3B and MHCC-97H cells were chosen for analysis. The authors first established stable ASPDH-overexpressing cell lines using Hep3B and MHCC-97H hepatocellular carcinoma cells. Quantitative analysis demonstrated a significant upregulation of ASPDH expression in the transfected cells compared to control groups, confirming successful generation of the overexpression models (Fig. 3A). Compared to the oe-NC groups, the proliferation, clone formation, migration, and invasion abilities of Hep3B and MHCC-97H cells in the oe-ASPDH group were significantly inhibited. This suggested that regulating ASPDH has an impact on the function of liver cancer cells (Fig. 3B‒E). To investigate the function of ASPDH in Hep3B and MHCC-97H cells, the authors knocked down ASPDH expression in Hep3B and MHCC-97H cells via siRNA transfection. qRT-PCR results showed that the mRNA expression levels of ASPDH in the si-ASPDH#1 and si-ASPDH#2 groups were significantly lower compared to the si-NC group (Fig. S1A), indicating successful transfection efficiency and effective silencing of ASPDH. In the cell proliferation assay, CCK-8 analysis revealed that the proliferative capacity of cells in the si-ASPDH#1 and si-ASPDH#2 groups was significantly higher than that in the si-NC group at 12 h, 24 h, and 48 h (Fig. S1B). The EdU assay further confirmed that cell viability was markedly increased after ASPDH knockdown (Fig. S1C). Regarding migration and invasion capabilities, Transwell assay results showed that the numbers of migrating and invading cells in the si-ASPDH#1 and si-ASPDH#2 groups were significantly higher than those in the si-NC group (Fig. S1D). In conclusion, ASPDH knockdown significantly enhances the proliferation, migration, and invasion abilities of Hep3B and MHCC-97H cells. In summary, overexpression of ASPDH effectively inhibited the proliferation, invasion, and migration of Hep3B and MHCC-97H cells.

### ASPDH could negatively regulate lactate levels and PD-L1 expression

**Fig. 4:**
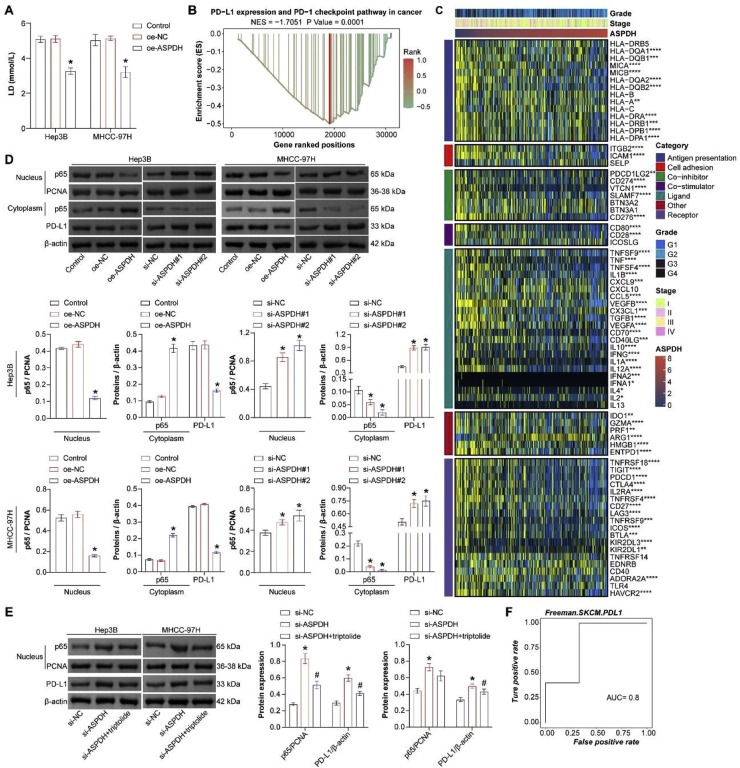

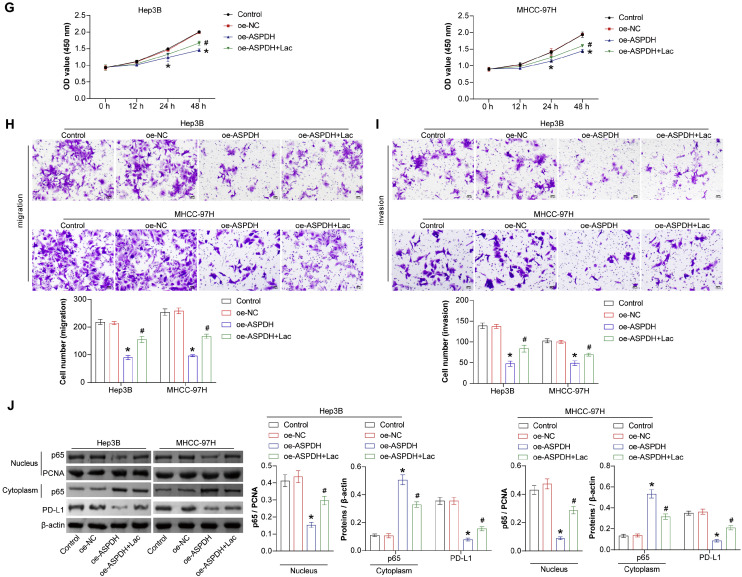
ASPDH could inhibit lactate levels and PD-L1 expression. (A) The detection of lactate levels. (B) GSEA visualization of PD-L1 expression and the PD-1 checkpoint pathway in cancer. (C) Heatmap showing the correlation between immune checkpoint and ASPDH. (D) Western blot was used to analyze p65 (nuclear), p65 (cytoplasm), and PD-L1 expression. (E) Western blot was used to analyze p65 (nuclear) and PD-L1 expression. (F) ROC curve. (G) CCK-8 analysis of cell proliferation. (H and I) Analysis of cell migration and invasion. (J) Western blot detection of PD-L1 expression and PD-1 checkpoint pathway in cancer signaling pathway-related indicators (p65 (nuclear), p65 (cytoplasmic), PD-L1). * Indicates a significance level of p < 0.05 compared to the oe-NC group.

The above results show that the lactate-related gene ASPDH can intervene in the function of liver cancer cells. Further investigation into its effect on lactate secretion is warranted. Lactate assay kit detection revealed a significant decrease in lactate secretion in Hep3B and MHCC-97H cells overexpressing ASPDH. Thus, ASPDH can inhibit lactate secretion (Fig. 4A). ASPDH was negatively correlated with immune checkpoint CD274 (PD-L1) (Fig. 4C). The GSEA analysis results showed that ASPDH inhibited PD-L1 expression and PD-1 checkpoint pathway in cancer (NES = −1.7051, p = 0.0001) (Fig. 4B). A study has shown that activating p65 could promote the expression of PD-L1.^16^ Therefore, quantitative analysis of PD-L1 immune checkpoint signal pathway-related indicators, p65, and PD-L1, was conducted. In the cell nucleus, the expression of p65 decreased after ASPDH overexpression, while it increased in the cytoplasm. PD-L1 expression decreased in the cytoplasm after ASPDH overexpression. Compared to the si-NC group, the expression of p65 and PD-L1 in the nucleus increased, while the expression of p65 in the cytoplasm decreased in Hep3B and MHCC-97H cells in both the si-ASPDH#1 and si-ASPDH#2 groups (Fig. 4D). The experimental results demonstrated that in Hep3B and MHCC-97H cells, ASPDH knockdown significantly upregulated the expression levels of nuclear p65 and PD-L1, whereas this upregulation was partially reversed upon combined treatment with the NF-κB inhibitor triptolide (50 nM). Specifically, compared to the si-NC group, the si-ASPDH group exhibited markedly increased nuclear p65 and PD-L1 expression. In contrast, the si-ASPDH + triptolide group showed reduced expression of both proteins relative to the si-ASPDH group, though levels remained higher than those in the si-NC group. These findings confirm that ASPDH positively regulates PD-L1 expression by modulating p65 nuclear translocation, suggesting that the NF-κB pathway plays a critical role in ASPDH-mediated PD-L1 regulation (Fig. 4E). The ROC curve analysis of PD-L1 demonstrated an AUC value of 0.8, suggesting that ASPDH expression may be associated with patient response to PD-L1 immunotherapy (Fig. 4F). Lactate levels and PD-L1 expression decreased in Hep3B and MHCC-97H cells after ASPDH overexpression.

To further investigate the role of lactate in ASPDH-mediated biological effects, the authors conducted sodium lactate rescue experiments in ASPDH-overexpressing cells. CCK-8 cell proliferation analysis revealed that compared with the oe-ASPDH group, the oe-ASPDH+Lac group showed a significant increase in cell proliferation capacity, although the proliferation level remained lower than that in the Control and oe-NC groups (Fig. 4G). Similarly, cell migration and invasion assays demonstrated that exogenous sodium lactate treatment partially reversed the inhibitory effect of ASPDH overexpression on the migration and invasion abilities of hepatocellular carcinoma cells (Fig. 4H and 4I). Furthermore, Western blot analysis indicated that after sodium lactate treatment, the expression levels of nuclear p65 and PD-L1 increased compared to the oe-ASPDH group but remained lower than those in the Control and oe-NC groups; conversely, the expression of cytoplasmic p65 showed a corresponding decreasing trend (Fig. 4J). These results collectively suggested that lactate is involved in the regulatory process of ASPDH on NF-κB activation, PD-L1 expression, as well as the capacities for proliferation and migration.

### Drug sensitivity prediction

**Fig. 5:**
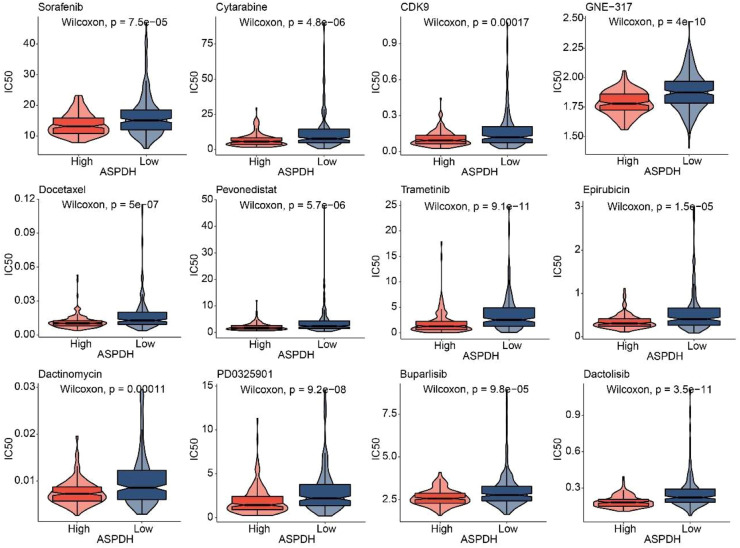
ASPDH could affect drug sensitivity in patients.

Whether the expression of the lactate-related gene ASPDH is related to drug sensitivity is worth further study. The oncoPredict prediction revealed that patients with high ASPDH expression exhibited higher sensitivity to 12 drugs, including Sorafenib, Cytarabine, CDK9, GNE-317, Docetaxel, Pevonedistat, Trametinib, Epirubicin, Dactinomycin, PD0325901, Buparlisib, and Dactolisib (Fig. 5). In conclusion, overexpression of the lactate-related gene ASPDH can enhance drug sensitivity in liver cancer patients.

### Somatic mutation analysis

**Fig. 6:**
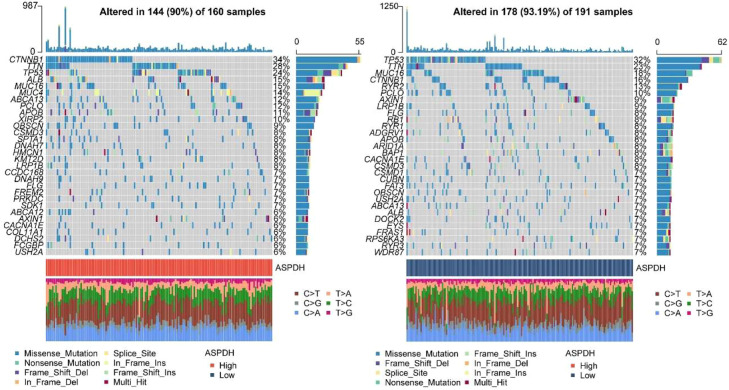
ASPDH is associated with somatic mutations in patients with liver cancer.

The above results showed that liver cancer patients with high expression of ASPDH are more sensitive to drugs. Therefore, the authors wanted to further analyze the differences at the gene level. Results showed that under the high expression of ASPDH, these genes had a higher mutation rate, such as CTNNB1 (34%), TP53 (24%), MUC16 (15%), and ALB (15%), among others. The main mutation types were Missense_Mutation, Frame_Shift_Del, and In_Frame_Del. However, under a low-ASPDH, these genes exhibited not only Missense_Mutation but also more Nonsense_Mutation. This analysis revealed several gene mutations associated with differential ASPDH expression levels in liver cancer, including TP53, TTN, and MUC16. Regarding mutation burden, genomic alterations were detected in 144 of 160 samples (90%) from patients with high ASPDH expression, compared to 178 of 191 samples (93.19%) in the low ASPDH expression group (Fig. 6). In conclusion, ASPDH may be associated with somatic mutations in patients with liver cancer.

### Overexpression of ASPDH inhibits the development of liver cancer

**Fig. 7:**
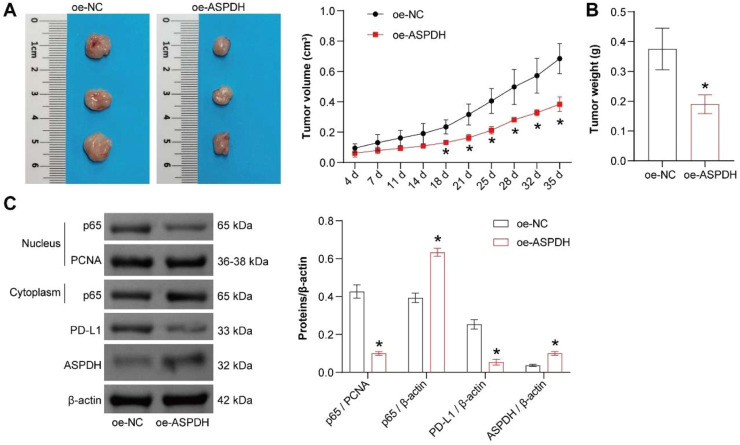
Overexpression of ASPDH inhibits the development of liver cancer. (A) Tumor image and volume. (B) Tumor mass. (C) Western blot was used to analysis of p65 (nuclear), p65 (cytoplasm), ASPDH and PD-L1 expression. * Indicates a significance level of p < 0.05 compared to the oe-NC group.

To investigate the impact of ASPDH overexpression on the *in vivo* growth of Hep3B cells, the *in vivo* experimental results demonstrated that, compared to the oe-NC group, the oe-ASPDH group exhibited a significantly slower tumor growth trend (Fig. 7A). Furthermore, tumor photographs and weight measurements revealed a notable reduction in both tumor volume and mass in the oe-ASPDH group (Fig. 7B). Western blot analysis further confirmed that ASPDH protein expression was significantly upregulated in the oe-ASPDH group, accompanied by increased cytoplasmic p65 levels, while nuclear p65 and PD-L1 expression were markedly downregulated (Fig. 7C). These findings suggested that ASPDH overexpression may suppress liver cancer by inhibiting p65 nuclear translocation and its downstream PD-L1 expression. The results align with the initial hypothesis, indicating that ASPDH holds potential therapeutic value for Liver cancer treatment.

## Discussion

In this study, the authors established a prognostic risk model based on 320 lactate metabolism-related genes, identifying four core genes (LPCAT1, TMEM220-AS1, ASPDH, and LECT2) with clinical significance. Notably, hepatocellular carcinoma patients with low ASPDH expression exhibited significantly worse survival outcomes. Mechanistically, ASPDH showed a strong negative correlation with immune checkpoint PD-L1 and functioned as a tumor suppressor; its overexpression not only inhibited proliferation in Hep3B and MHCC-97H cells but also concurrently reduced lactate production and PD-L1 expression.

Lactate also regulates the metabolism of innate and adaptive immune cells, including T-cells, Natural Killer (NK) cells, Natural Killer-T (NKT) cells, and dendritic cells, by inhibiting the function of CD8+.^17^ Liver cancer patients with a high LM Enrichment score had a poorer prognosis. A further screening of the lactate metabolism-related gene cluster identified four genes: TMEM220-AS1, LECT2, and ASPDH. Notably, although the specific mechanisms of these genes in liver cancer remain incompletely understood, three of them (LPCAT1, TMEM220-AS1, and LECT2) have been reported in liver cancer, with their aberrant expression affecting patient survival.^18^, ^19^, ^20^ In liver cancer xenograft models, LECT2 was shown to suppress tumor growth by inhibiting angiogenesis.^21^ The lncRNA TMEM220-AS1 was found to inhibit liver cancer cell proliferation and invasion by regulating the TMEM220/β-catenin axis.^22^ Similarly, LPCAT1 was demonstrated to promote the malignant transformation of liver cancer cells through direct suppression of STAT1.^23^ Survival analysis showed low ASPDH correlated with poor prognosis (GSE54236, ICGC, and GSE116174), suggesting context-dependent roles. PD-L1 demonstrated strong diagnostic value (AUC = 0.8), supporting its biomarker potential. The present survival analysis was consistent with these findings: high expression of LPCAT1 was associated with poor prognosis in liver cancer patients, while low expression of TMEM220-AS1, LECT2, and ASPDH also correlated with unfavorable outcomes.

The present functional studies demonstrated that ASPDH overexpression was sufficient to elicit a significant decrease in lactate secretion in Hep3B and MHCC-97H cells, indicating that ASPDH consequently acts as a potent negative regulator of lactate production. As a key enzyme in aspartate metabolism, ASPDH overexpression likely alters the intracellular metabolic flux of aspartate. This can be broken down into two aspects: Pyruvate, the end product of glycolysis, is the direct substrate for Lactate Dehydrogenase (LDH) to generate lactate. ASPDH overexpression may enhance the conversion of aspartate into other metabolic pathways, such as oxaloacetate. This process could indirectly consume pyruvate or compete for intracellular reducing equivalents (NADH), thereby reducing the pyruvate pool available for lactate production.^9^ ASPDH may promote lactate reuse through anaplerosis that reinforces the TCA cycle. Aspartate metabolism is closely linked to the TCA cycle. Increased ASPDH activity likely elevates levels of TCA cycle intermediates. This not only boosts mitochondrial oxidative phosphorylation capacity ‒ shifting cells from the glycolysis-dependent Warburg effect to a more efficient energy production mode ‒ but may also enhance the cell's ability to reuptake existing lactate via Monocarboxylate Transporters (MCTs). This lactate can then be converted back to pyruvate via the reverse LDH reaction and enter the TCA cycle, thereby promoting lactate “reuse” .^8^ The observed decrease in lactate secretion is more likely a result of the combined action of “inhibited glycolytic flux” and “promoted lactate reuse”, rather than either mechanism alone.

The experimental results of this study lean more toward supporting the notion that “promoting lactate recycling” plays a critical role. The most direct evidence comes from the functional rescue experiment: exogenous lactate supplementation can partially reverse the inhibitory effects of ASPDH overexpression on hepatocellular carcinoma cell proliferation, migration, invasion, and the p65/PD-L1 signaling pathway. If the role of ASPDH were merely unidirectional in inhibiting glycolytic flux and reducing lactate production, then supplementing lactate as an end product should not effectively restore these cellular functions. Conversely, the effective “rescue” by lactate strongly suggests that ASPDH overexpression may create a metabolic environment in which cellular utilization of lactate or lactate-mediated signaling becomes essential. This aligns well with the hypothesis that “ASPDH promotes lactate entry into the TCA cycle as an anaplerotic substrate”. When ASPDH is upregulated, cells may become more reliant on lactate as a carbon source, leading to the sequestration and consumption of endogenous lactate and a consequent reduction in its secretion. Once exogenous lactate is supplemented, cellular metabolism and functions are partially restored. Additionally, ASPDH overexpression suppresses the nuclear translocation of NF-κB, a key pro-inflammatory and pro-survival transcription factor. Given that NF-κB signaling has been widely reported to upregulate glycolysis^24^ related genes and promote metabolic reprogramming akin to the Warburg effect,^25^ the observed inhibition of NF-κB activity in this study is phenotypically consistent with reduced lactate secretion. Together, these findings support the hypothesis that ASPDH may drive metabolic remodeling.

In recent studies, it was found that lactate derived from tumors could induce the expression of PD-L1 through the NF-κB/COX-2 pathway. Furthermore, lactate-H+ could prolong the lifespan of neutrophils, partially promoting PD-L1 expression.^26^ Mechanistically, it increased nuclear p65 and PD-L1 while reducing cytoplasmic p65. NF-κB inhibition (triptolide) reversed these effects, confirming ASPDH regulates PD-L1 via p65 nuclear translocation. The *in vivo* xenograft model demonstrated that ASPDH overexpression attenuated tumor growth, coinciding with reduced nuclear p65 and PD-L1 levels. This supports the *in vitro* data, positioning ASPDH as a negative regulator of the NF-κB/PD-L1 axis. The concordance between cytoplasmic p65 accumulation and PD-L1 downregulation further implies that ASPDH sequesters p65 in the cytoplasm, limiting its transcriptional activity. The GPR81 receptor pathway is one important potential mechanism. Lactate is a natural agonist for GPR81. In the tumor microenvironment, binding of lactate to GPR81 on the surface of tumor cells or immune cells can trigger G protein-coupled signaling cascades, ultimately leading to the release and nuclear translocation of the p65 subunit.^27^ The recently identified histone lactylation modification offers another direct epigenetic regulatory mechanism. Lactate can serve as a precursor for the covalent modification of histones (*e.g.*, H3K9la). This modification typically occurs at gene promoter regions and is associated with gene activation.^28^ Therefore, lactate, likely by increasing the intracellular lactate concentration, may directly enhance the level of histone lactylation at the promoters of NF-κB target genes (such as PD-L1), thereby upregulating their transcription. This process can even occur independently of the canonical NF-κB nuclear translocation. In the present study, the authors attributed the observed effects to the nuclear translocation of NF-κB/p65. However, this is likely a downstream manifestation resulting from the upstream events described above, such as GPR81 signaling or increased transcriptional activity driven by lactylation. The observed accumulation of p65 in the nucleus could be a direct consequence of GPR81 pathway activation, or it could represent a feedback response following the increased transcription of genes like PD-L1 due to lactylation, potentially aimed at maintaining cellular homeostasis. The authors acknowledge that attributing the phenomenon primarily to NF-κB activation without fully elucidating the specific upstream mechanisms is a limitation of the present study. The partial rescue of the phenotype by exogenous lactate could be because the supplemented concentration did not fully reconstitute the endogenous lactate microenvironment, or because GPR81 signaling and lactylation modification play collaborative or distinct roles in regulating NF-κB. In order to clarify the upstream mechanisms by which ASPDH regulates p65 nuclear localization, future studies should focus on distinguishing the respective roles of GPR81 receptor signaling and histone acetylation. A key experimental direction is to modulate ASPDH while separately or simultaneously blocking GPR81 signaling, lactate metabolism, and histone lactylation, followed by systematic analysis of changes in p65 nuclear translocation. This strategy will effectively determine whether ASPDH regulates NF-κB activity by influencing the lactate-GPR81 axis or by altering the epigenetic landscape, thereby uncovering its intrinsic pathway of action and transforming the current correlative observations into causal mechanistic evidence.

Although the present study demonstrates that ASPDH significantly influences lactate metabolism and cancer progression, the authors acknowledge that its broader metabolic roles and potential compensatory mechanisms remain to be fully elucidated. Previous studies have shown that when glycolysis or lactate-related pathways are disrupted, cancer cells can activate alternative metabolic pathways, such as glutaminolysis,^29^ fatty acid oxidation,^30^ and serine/glycine metabolism.^31^ While these current findings primarily focus on lactate metabolism, ASPDH may also interact with other metabolic networks, including the pentose phosphate pathway, lipid biosynthesis, and mitochondrial redox balance, which could influence therapeutic outcomes. Additionally, compensatory adaptations over time may mitigate the effects of ASPDH inhibition, as observed with other metabolic interventions.^32^ Finally, the absence of direct ASPDH enzymatic activity measurements due to the lack of a commercial assay also constitutes a limitation, and future efforts will be directed toward establishing a robust method to confirm its biochemical function. Future studies will employ systems-level metabolomics and long-term perturbation experiments to uncover these potential adaptations and determine whether ASPDH’s role varies across cancer types with distinct metabolic dependencies.

In terms of drugs, the sensitivity of 12 drugs Sorafenib, Cytarabine, CDK9, GNE-317, Docetaxel, Pevenastat, Trametinib, Epirubicin, Dactinomycin, PD0325901, Bupalicib and Dactolisib were predicted based on the expression level of ASPDH. Among them, Sorafenib,^33^ Cytarabine,^34^ CDK9,^35^ Docetaxel,^36^ Trametinib,^37^ Epirubicin,^38^ and PD0325901^39^ have been reported to be available for the treatment of liver cancer. GNE-317 is used in the treatment of glioblastoma.^40^ Dactinomycin is indicated for recurrent or persistent endometrial carcinoma.^41^ However, it is critical to note that Dactinomycin carries known risks of hepatotoxicity, which raises serious safety concerns regarding its potential application in liver cancer patients.^42^ Bupalicib has been associated with breast cancer treatment.^43^ Dactolisib demonstrates potential therapeutic efficacy against prostate cancer.^44^ Given the exploratory nature of these predictions, the drug list should be regarded not as direct therapeutic options but as candidates requiring thorough experimental validation. Notably, the therapeutic mechanisms of five agents ‒ GNE-317, Dactinomycin, Palbociclib, Dactolisib, and Pevonedistat ‒ in liver cancer remain poorly characterized, and future work must systematically evaluate both efficacy and toxicity through *in vitro, in vivo*, and mechanistic studies to assess their clinical translational potential.

## Conclusion

In conclusion, ASPDH, a lactate metabolism-related gene identified through bioinformatics screening, effectively inhibits the expression of PD-L1. After overexpression of ASPDH, secretion of Lactate decreased in Hep3B and MHCC-97H cells, and their proliferation, clonogenicity, migration, and invasion abilities diminished. ASPDH suppresses liver cancer progression by regulating lactate metabolism and the NF-κB/PD-L1 signaling pathway. These findings will help further understand the roles of some lactate metabolism-related genes in liver cancer.

## Data availability

The data supporting this study are included in the article.

## Ethics approval

This study was approved by the Medical Ethics Committee of Hunan Provincial People's Hospital (n° [2024]−69). The animal procedures conformed to the ARRIVE guidelines and related regulations.

## Consent to participate

Not applicable.

## Consent to publish

Not applicable.

## Funding

The authors declare that no funds, grants, or other support were received during the preparation of this manuscript.

## CRediT authorship contribution statement

**Ding Li:** Data curation, Formal analysis, Visualization, Methodology, Writing – original draft. **Muzi Li:** Data curation, Formal analysis, Visualization. **Mengfan Yan:** Data curation, Formal analysis. **Yuanyuan Xiong:** Data curation, Validation, Formal analysis, Writing – review & editing.

## Declaration of competing interest

The authors declare no conflicts of interest.

## Appendix Supplementary materials

Image, application 1
