# Supplementary material for: ASPDH inhibits the proliferation, migration, and invasion of liver cancer cells by regulating lactate metabolism and the NF-κB/PD-L1 pathway
Source: Clinics (Sao Paulo). 2026 May 13;81:100946. doi: 10.1016/j.clinsp.2026.100946 (PMC13195349; doi:10.1016/j.clinsp.2026.100946)

**CLINICS-D-25-00184**

**Supplementary Files**

**Supplementary Figure S1 Knockdown of ASPDH could promote the proliferation, invasion, and migration of liver cancer cells.** (A) The expression of ASPDH. (B) Cell proliferation. (C) EdU was ultized to analysis of cell proliferation. (D) Transwell analysis was utilized to measure cell migration and invasion. * Indicates a significance level of p < 0.05 compared to the si-NC group.


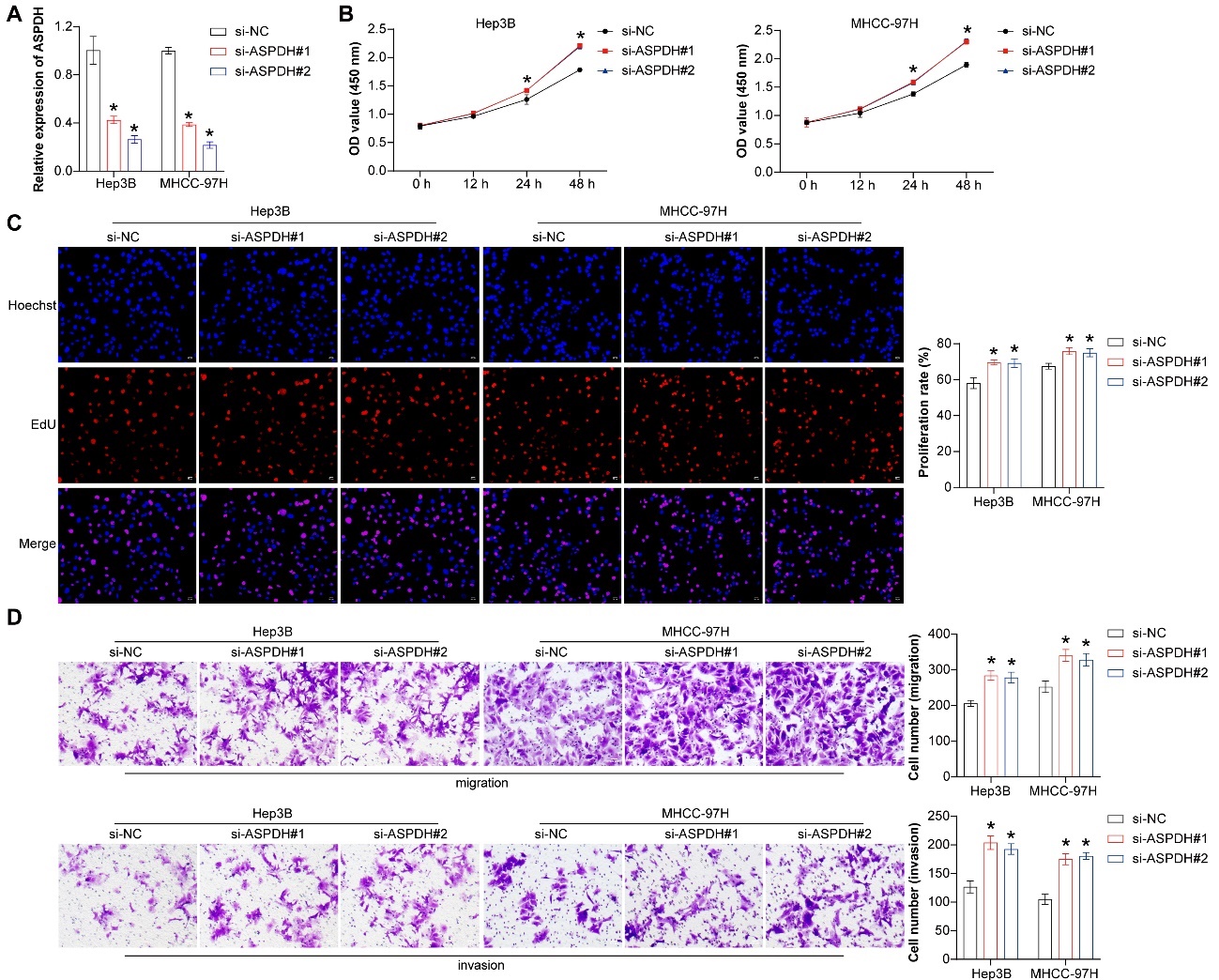

Supplement: Supplementary file 1 [file mmc1.docx]
